# OdorTAM: Technology Acceptance Model for Biometric Authentication System Using Human Body Odor

**DOI:** 10.3390/ijerph192416777

**Published:** 2022-12-14

**Authors:** Sameena Naaz, Sarah Ali Khan, Farheen Siddiqui, Shahab Saquib Sohail, Dag Øivind Madsen, Asad Ahmad

**Affiliations:** 1 Department of Computer Science and Engineering, School of Engineering Sciences and Technology, Jamia Hamdard, New Delhi 110062, India; 2 USN School of Business, University of South-Eastern Norway, 3511 Hønefoss, Norway; 3 Department of Management, School of Management and Business Studies, Jamia Hamdard, New Delhi 110062, India

**Keywords:** body odor, biometric authentication, technology acceptance model (TAM), privacy, security

## Abstract

Body odor is a biometric feature unique to each individual, and it can be used for authentication. However, decision makers must learn about the users’ level of acceptance of this technology, as well as their thoughts on the system’s features and procedures. In this study, a technology acceptance model (TAM) for body-odor-based biometric techniques named OdorTAM was proposed and validated. An English language questionnaire was developed in a web-based, easy-to-read format on Google Forms. The survey consisted of 19 questions, and 150 responses were received. Statistical analysis of the responses was carried out, and it was found that all the hypotheses were supported. Therefore, the OdorTAM model appears to be satisfactory. To this end, we posit that a body-odor-based biometric technique can be one of the alternatives for authentication, and it can also be used along with some other techniques for improved security. The study contributes to the literature on consumers’ understanding of biometric technologies, in particular odor detection, which has received relatively less attention in extant research.

## 1. Introduction

Each human being is distinct, and each of them can be defined by their intrinsic and behavioral characteristics. This is the groundwork for biometric verification. Biometric technologies can recognize people and their unique features from fingerprints [1,2], faces, DNA, signatures or irises [3,4,5,6]. A variety of biometric technologies, such as facial and voice recognition, can be combined to improve safety or reliability. With the advancements in sensor technology, another non-invasive biometric technique gaining importance is body odor [7]. Body odor recognition is a contactless physical biometric, which attempts to confirm an individual’s identification by analyzing the olfactory properties of the human body smell. Several researchers have described it as feasible for personal identification [8]. To identify an individual via their odor is a crucial task. To simplify the task, Wongchoosuk, Lutz and Kerdcharoen [8] have proposed an electronic nose to filter out the specific smell, which can be dynamic, and changes with the changing environments and conditions.

Odor can be used as an identification method either in combination with other biometric tools or separately. In fact, body odor as a biometric has been recently studied, and there is plenty of opportunity for researchers to explore. However, developing a complete system that is new requires a lot of investment in terms of resources and time. To this end, users’ perception identification and consensus building is an important aspect [9]. Therefore, it is necessary to consider the understanding and acceptance of this technology. User understanding and adoption of biometric technologies will significantly hinder or aid the product’s deployment [10,11,12]. Therefore, assessing and interpreting market opinion and adoption of biometric technology is essential, since even a highly advanced and sophisticated technological approach may be ignored, feared and discarded by end users. In 1985, Fred Davis proposed the technology acceptance model, also widely known as TAM, which would conclude that the user would accept or reject a model [13,14]. This model posits that the user’s motivation to use a particular technology depends on three factors: perceived ease of use, perceived usefulness and attitude toward using the technology [10]. Most users feel frightened, reluctant or uncomfortable about these systems, particularly as they perceive them as a means of potential privacy infringements. The feelings and expectations of such users increase the risk of rejection and may lead to failure in the implementation of such biometric technologies. Therefore, it is of vital importance to understand the various factors that influence the acceptance of biometric technology [15,16,17,18].

The consumers’ understanding of biometric technology has been discussed in various previous surveys, but odor detection is a type, which has received relatively less attention. In the present paper, we address the following overall research question: *How do biometric users perceive a biometric technique related to body odor?* We discuss odor scanning expectations and behavior and provide recommendations for research and practice. A survey was carried out with the technology acceptance model (TAM) as the research framework.

The topic of this paper should also be of interest to readers of the journal, since it can be linked to debates in *IJERPH* about biometrics-related issues [19,20,21] and the use of variations and modifications of TAM in the context of environmental and public health research [22,23,24,25].

The rest of the paper is organized as follows. Section 2 gives the details of related work already carried out in the area of biometric technology. It also provides an overview of the related work carried out on the application of TAM in different contexts. Section 3 and Section 4 discuss human body odor as a biometric technique and the privacy issues that come with it. Section 5 describes the OdorTAM model proposed in this work. The research methodology is presented in Section 6. Section 7 provides a descriptive statistical analysis, and Section 8 presents a detailed evaluation of the OdorTAM hypotheses. Section 9 provides the results of the regression analysis. Section 10 compares the results obtained in this work with some previous works carried out in the same area. Lastly, Section 11 concludes the paper by summarizing the main results and identifying the contributions, limitations and areas for future work.

## 2. Literature Review

In a study by Yang and Lee [26], it was found that compared with other biometric identifiers, such as iris, fingerprints and facial recognition, body odor has the lowest error rate (15%). The statistical approaches that are commonly used to determine human body odor (HBO) elements have been studied in Ref [27]. As a first step, they assessed the rising forms of BOs and the sampling and/or pre-concentration techniques. The authors of Ref [28] reveal the advantages of tackling the problems faced by law enforcement agencies, the framework for public protection and the national defense guarantee of the country. A paper by Khan and Naaz [29] discussed the application of different biometric techniques in the cyber security domain. This paper made a comparison between three different approaches, namely the human body odor biometric system, finger vein and iris, and discussed their benefits and limitations.

The acceptance of a new authentication technique by users has been studied in several previous studies. Although TAM is a highly cited and used model [30], the authors in Ref [31] observed that it lacks sufficient rigor and relevance to make it an established theory for the IS community. Several researchers have criticized the TAM model for not considering relevant factors, such as hedonism, time, space and trust, which may play a relevant role in determining the behavioral intentions of consumers. The new standards for perceived usefulness (PU) and perceived ease of use (PEOU) were validated and developed in Ref [29]. The authors performed a study of 152 users to find that efficiency has a significantly stronger connection with a system’s use activity relative to perceived user friendliness. The authors Khan and Naaz [29] established that both mechanisms of social control (subjective standard, voluntariness and image) and cognitive instrumental mechanisms (relevance of jobs, consistency of performance, demonstrability of outcomes and perceived ease of use) had a major impact on user acceptability. The usage of biometrics in the E-government systems is examined and addressed in Refs [32,33]. TAM and other theoretical paradigms have been studied for different domains by several authors [34,35,36,37]. The authors in Ref [38] carried out a survey of 84 participants to test the understanding and recognition of the usage of odor as an authentication method. In their findings, they revealed how many people did not know about this method, and how they perceived it as being dangerous in terms of safety.

The TAM model has been studied for biometric authentication by the author in Ref [7], where trust was added as one of the factors along with PU and PEOU. TAM has also been used in other contexts, such as sports branding, evaluation of online video usage and in education [39,40,41]. Additionally, many other studies apply TAM [42,43,44]. Biometric methods in various categories, such as behavioral and biological, have been discussed by the authors in Ref [45].

## 3. Human Body Odor as a Biometric Technique

Human olfaction is one of the unique senses that humans possess, which provides information about the distinct chemicals from remote origins and distant sources in real time [27]. Human beings emit a very complex range of molecules. They could be non-volatile or volatile. This emission of molecules depends on the immune system, genetics, environment, diet and stress.

**Figure 1:**
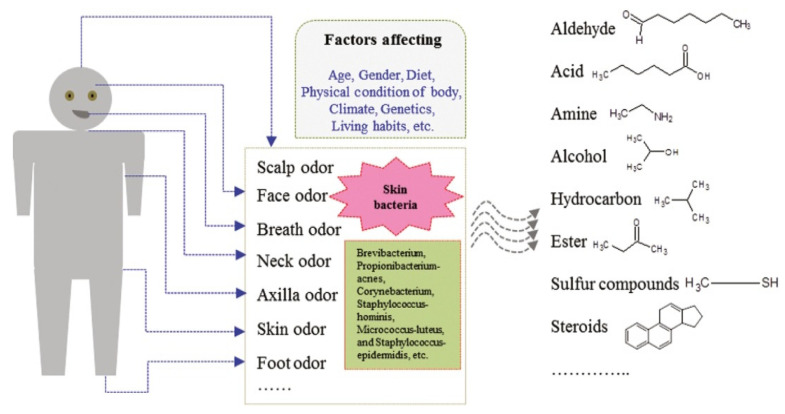
Different odor-producing spots in the human body (source: Ref. [46]).

These volatile compounds are emitted through various areas of the human body, as shown in Figure 1. The human body odor (BO) study may be classified into three groups, depending on the standard parameters extracted from the literature survey:

1. Perspiration (skin odor),
2. Odor released from the oral cavity (exhaled breath),
3. Odor released from human excreta (urine).

BO is the foul and irritating scent produced on the skin by a combination of sudor (sweat) and bacteria [47]. The most common depiction of the production of human odors is the bacterial action and activities on dead cells of the skin. Despite popular belief, sweat is an odorless secretion of the body. However, the action and multiplication of bacteria on the human skin led to the breaking down of these secretions, with strongly unpleasant and highly disagreeable odors.

The study of the analysis of the physical properties of living organisms is biometrics [48]. Although several biometric modalities have been studied well in the past, very little is known about the body odor biometric system. The principle of BO biometric systems is based on the fact that virtually each person’s smell is unique and distinctive. The captivation of this unique smell is obtained through sensors from non-intrusive body parts, such as the armpits, the back of the hands, etc. BO recognition is basically a physical but contactless biometric, which attempts to identify the claimed identity by analysis of the olfactory properties of human scent [49]. The key aim of human odor detection is to construct an artificial device as reactive as possible. In the human olfactory model, such electronic device is believed to be developed. Therefore, the human olfactory pattern must be thoroughly comprehended before constructing this device [50].

### Quantitative Analysis of Odor

A variety of quantitative approaches can be used to evaluate different types of odors, including concentration and evident severity measurement.

Odor Concentration: The odor concentration is measured using an olfactometer test. A diluted odorous mixture and an odorless gas are fed separately to a group of e-noses from the sniffing ports kept in a mild odor space during the olfactory testing procedure. A comparison of the gases emitting out of each of the sniffing ports is made, after which, the existence of odor is determined. Then, a balance of two chemicals reduces the gas-diluting ratio concentration increases by an average of two. This particular procedure is continuously repeated and will continue for a number of levels of dilution [51]. The e-nose responses over a variety of dilution settings are used to measure the odor concentration according to the European Odour Units (ouE/m^3^). The key gas calibration panel used is Butan-1-ol, which gives 1 ouE/m^3^ at a certain dilution. The concentration is represented as the dilution required to reach a standard for panel detection. Mathematically

C=(Vo+Vf)/Vo

where *C* is the concentration of the odor, *Vo* is the volume of the odorous sample, and *Vf* is the quantity of odorless air needed to reach the threshold.

Odor Intensity: The force of odor is the subjective frequency of the sense of scent. This strength property is used to identify the source of the odor and may be more specifically correlated with odor nuisance. The subjective intensity of the sense of odor is calculated along with the concentration of odor. This can be modeled according to the Weber–Fechner law [52].

I=a*log(c)+b

where “*I*” is the psychological intensity perceived at the dilution step of the butanol scale—*a* Weber–Fechner coefficient; “*c*” is the chemical concentration; and “*b*” is the constant of interception (0.5). Odor intensity can be measured using a scale of odor severity—a verbal definition of a sense of odor to which a numerical measure is given. The odor level can be classified by severity into the following: 0—No odor; 1—Very faint (Odor threshold); 2—Low; 3—Distinct; 4—Strong; 5—Very strong; 6—Intolerable.

## 4. User Privacy Concerns in Human Body Odor Technology

The human body odor can be determined from many areas: skin odor, body odor, mouth odor, urine odor, excreta odor, feet odor, etc. All of these can be determined through different means and can determine many factors about the person, especially medical. Capturing foot odor, urine odor, excreta odor, skin odor, etc., requires individual participation, and studies of these odors are mostly related to finding out about the health of a person. However, for non-intrusive biometrics, it is possible to capture body odor, which also includes odor from breath. As much as biometrics are developed and invented for public facilities, they can also hinder one’s privacy if not used ethically and morally. People nowadays are more aware of and concerned about their personal data and privacy. Government laws have also provided people with the power to make claims against any entity that they feel has intruded in their personal matters. Especially with a biometric technique like body odor biometrics, there are many potential privacy concerns.

Some of the findings about human body odors that can be a problem for the consumers of this biometric technology could be:

- Analyzing body odor can determine a person’s health. People tend to accept these diagnostic techniques for health and diseases, especially in the medical fields, food industry, environment industry or even pharmaceutics. However, in the case of authentication, people might not want to disclose information about their health and diseases. Analyzing body odor could determine diseases, such as diabetes, cancer, lung carcinoma, hormonal imbalances, chronic obstructive pulmonary disease(COPD), liver diseases, metabolic disorders, etc. [53].
- Body odor can also be a determinant of certain types of foods and drinks one is consuming or has recently consumed. For example, sulphureous foods can contribute to body odor. For many ethical reasons, people would like to keep this information personal.
- Capturing of the body odor can also detect if a person is under stress [54].
- It is also possible to determine what medications a person is on. Many analgesic pain killers, SSRI antidepressants, contraceptive medications and heart-functioning-based narcotics include persistent sweating as part of their documented list of side effects.
- Body odor analysis is also capable of determining the recent activities one has been involved in (e.g., sexual intercourse). There is a risk that this type of information could be used in an unethical way against the consumers without their knowledge.
- Human body odor is also capable of determining the emotions of a person. Researchers have found that feelings, such as terror, anger, disgust or happiness, may be "smelled" by people through excreted chemical signals [55,56,57].

## 5. Proposed Solution: OdorTAM

Although biometric security has gained acceptance these days, it still has several limitations, such as variation in the biometric signals due to environmental conditions and differences in sensors. Another important problem is that people feel that these devices are very intrusive, since they capture highly personal information, which could be very dangerous if they fall into the wrong hands. Body odor as a biometric authentication has been used for a long time by the police who use bloodhound dogs to identify culprits using their personal odor [45].

**Figure 2:**
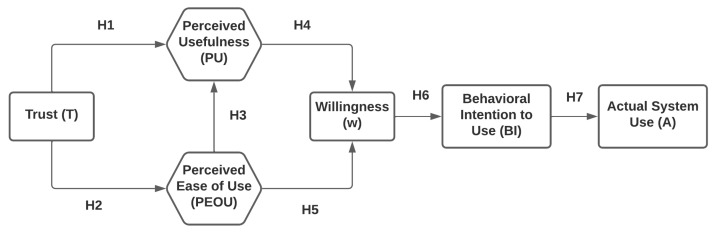
Proposed OdorTAM.

However, it is very essential to study the public acceptance of any technology from the point of view of stakeholders before investing in it. The OdorTAM model proposed in this work is depicted in Figure 2, and it tries to study the perceived usefulness and perceived ease of use of the body-odor-based biometric authentication by adding two external factors: trust and willingness. Willingness usually suggests attendant potential actions [58]. These factors have an impact on the intention to use the device, which in turn influences the actual use of the system.

### Hypotheses

Trust is undoubtedly the most powerful mechanism for mitigating uncertainty, a sense of risk and a sense of safety, and customer trust is assumed to play a pivotal role in the decisions of customers to adopt a biometric system by minimizing perceived risks and the confusion associated with accepting it. Any new technology can survive on the market only if it can be trusted and people are willing to use it [59,60]. Keeping in mind the relevance of trust in the acceptance of any technology, several researchers have studied the impact of trust in a certain technology on the usage intention [61,62,63]. TAM relies and focuses on PU and PEOU to describe and analyze the user acceptance of any technology. Another important factor that determines acceptance is the behavioral intention to use that technology. Based upon these factors, the following hypotheses were formulated for our proposed model.

**Hypothesis 1 (H1).**  *Customer trust and faith in technology has positive consequences and impact on perceived usefulness (PU).*

**Hypothesis 2 (H2).**  *High level of trust will lead to increased perceived ease of use (PEOU) of a biometric system.*

**Hypothesis 3 (H3).**  *Perceived ease of use (PEOU) has a positive influence on the perceived usefulness (PU).*

**Hypothesis 4 (H4).**  *Perceived usefulness will result in willingness (w) of the people to use the system.*

**Hypothesis 5 (H5).**  *Perceived ease of use (PEOU) will also result in people being willing to use the system, and hence, more willingness (w).*

**Hypothesis 6 (H6).**  *If people are willing to use the system, then their behavioral intention (BI) will also be positive.*

**Hypothesis 7 (H7).**  *Behavioral intention (BI) of users results in increase in actual usage of the system.*

## 6. Research Methodology

To understand the user acceptance and perception about the human body odor biometric systems, an English language questionnaire was developed in a web-based, easy-to-read format on Google Forms. Since TAM (proposed by Davis) is a reliable barometer for determining user acceptance and perception for any technology, the questionnaire was developed bearing in mind the key concepts of TAM and biometrics and the factors that impact consumer perception and intention to use a newly developed or recently introduced technology. The survey questionnaire was developed taking cue from the extant literature [13,64,65].

The survey was circulated via email and different social media platforms (WhatsApp Messenger and Instagram) to people from all age groups. Earlier researchers have suggested that, at times, the respondents’ responses are based on no actual use or on the little knowledge they have, impacting the validity of a study [66]. Keeping in mind the awareness of the biometric technologies and their acceptability, the study focused on college students and workers. A brief introduction of the HBOBT and the purpose of the study were provided to the respondents before the actual questionnaire. The questionnaire comprised a total of 18 questions. Out of these, four questions were about personal information, i.e., name, age, gender and profession. The other 14 questions were about the human body odor biometric system. Seven of these questions were designed using a 4-point Likert scale with values lying in the range of very likely to very unlikely. A total of 150 usable responses were received.

## 7. Descriptive Statistics Analysis

Of the total 150 respondents, 85 (56.7%) were male, 63 (42%) were female, and the remaining 2 (1.3%) specified their gender as “other”. Responses were received from people of all age groups, with most responses coming from students younger than 25 (66%), followed by the age group 26–50 (27.3%). A total of 5.3% of the respondents were in the age group 51–75, and we even had one response from an individual above 75 years of age. As far as their profession is concerned, 37.3% were students; 18% had a technology background, which included software developers, web developers, data analysts, risk managers, professors, engineers, operation executives, system engineers, data scientists, etc.; and 44.6% were from a non-technology-based background, which included doctors, homemakers, directors, music composers, professional artists, pharmacists, beauty bloggers, entrepreneurs, architects, chartered accountants, etc.

**Figure 3:**
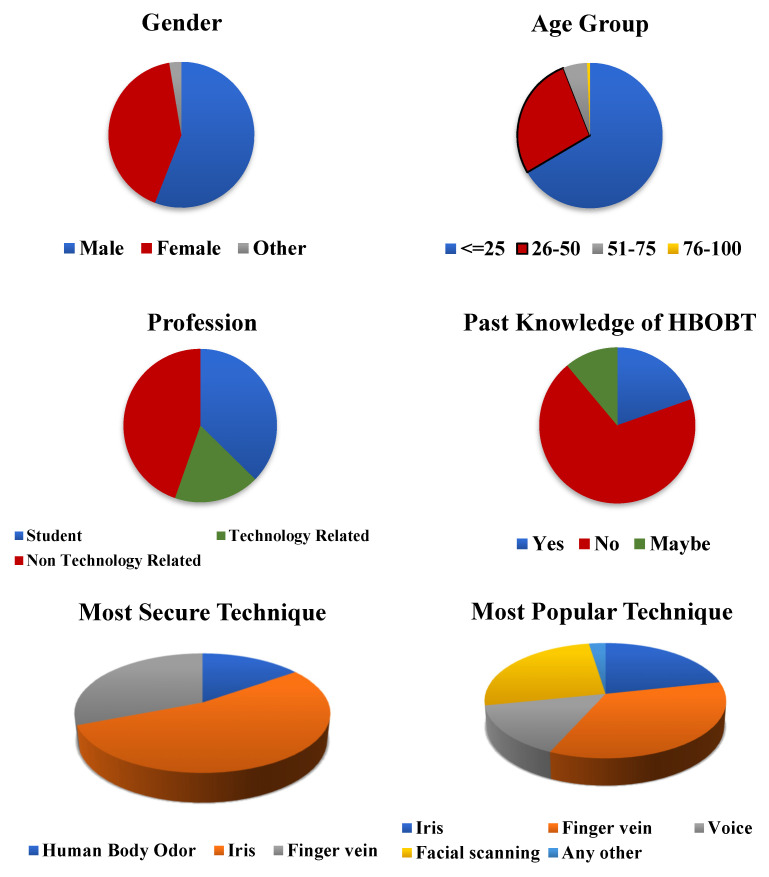
Distribution of personal and general information.

The respondents were asked whether they have heard about the human-body-odor-based biometric system, and most of them (70.7%) answered “no”. Only 20% were aware about this technology, and the remaining 11.3% were not sure about it. They were also asked, “Which biometric technology have you seen or used the most in your surroundings?”, and their response distribution is depicted in the pie chart in Figure 3. When asked about the security aspect of the different biometric techniques, most of the respondents replied in favor of the iris scan (66%), and a smaller portion (18.6%) considered body odor to be a reliable method from a security point of view. The distribution of personal and general information is depicted in Figure 3 and is also given in the tabular form in Table 1 and Table 2 contains TAM related responses.

## 8. Evaluation of OdorTAM

The mean and standard deviation of all the responses related to technology acceptance are presented in Table 3. Cronbach’s α analysis is a statistical technique, which is used to measure the internal consistency of data and tells us how reliable our system will be. As can be seen from Table 3, Cronbach’s alpha value for all the surveyed dimensions is greater than 0.7, except for trust (0.59) and behavioral intention to use (0.56). The reason for people not trusting the technology could be that they are not aware of it. This is apparent from the response to the question, “Did you know about HBOBT in the past?”, for which only 20% said “yes”. Most of the respondents (70.7%) were not aware of body odor as a biometric technique, and 11.3% were not sure about it. The Cronbach alpha values of almost all the variables were found to be around 0.7, thereby indicating that the scale was reliable [67].

Correlation coefficients are indicators of the strength of the linear relationship between two different variables. The correlation values indicate that the measures functioned effectively, and the proposed study model was satisfactory. A correlation analysis in the current study was carried out to evaluate the strength and directionality of the factors used in the OdorTAM model. The correlation statistics for all the factors of the OdorTAM are depicted in Table 4. It can be observed that perceived usefulness (PU) has a very high correlation (0.93) with perceived ease of use. The correlation between trust and perceived usefulness was found to be 0.80. Similarly, a correlation value of 0.78 was found between trust and PEOU. If we look at the correlation of willingness with behavioral intention, it was found to be 0.60, and that between behavioral intention and actual use was found to be 0.53.

## 9. Results of Regression Analysis

**Figure 4:**
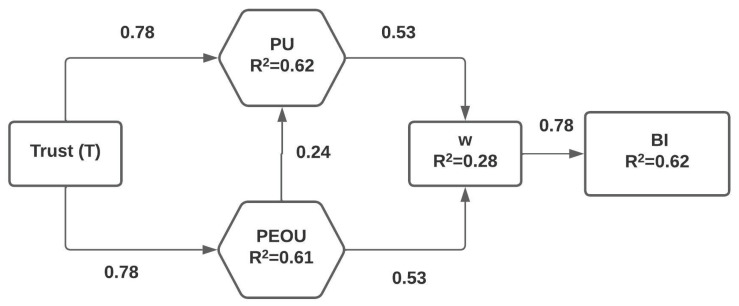
Path coefficients for the extended TAM model: Value on the path is the standardized coefficient (Beta); R^2^ is the coefficient of determination; and the significance value is less than 0.001.

The relationship between the independent variables and the dependent variable was studied using regression analysis. The relationships between all the TAM variables—Trust (T), Perceived Ease of Use (PEOU), Perceived Usefulness (PU), Willingness (w), Behavioral Intention (BI) and Actual System Use—were explored. The relationships for all the seven hypotheses were examined (see Figure 4). The quality of the model was measured using the standardized path coefficients (Beta), unstandardized coefficients (B and standard error), coefficient of determination (R^2^), t value and the significance level.

The results of the regression analysis show that the *p*-value corresponding to all variables is less than 0.001, which means that there exists a strong relationship between dependent and independent variables for all the cases. It can be seen from the obtained results that Trust has a strongly positive effect on Perceived Usefulness (PU) (β = 0.78, t = 15.47, *p* < 0.001), and hence, we can say that Hypothesis 1 is supported. The second hypothesis says that a high level of Trust will lead to increased Perceived Ease of Use (PEOU) of a biometric system, and from the regression parameters obtained (β = 0.78, t = 15.40, *p* < 0.001), it is evident that this hypothesis is also supported. The independent variables Trust and Perceived Ease of Use (PEOU) positively influence the dependent variable Perceived Usefulness (β = 0.78 and 0.24, t = 8.83 and 6.96, *p* < 0.001), and hence, Hypothesis 3 is also strongly supported. The results for prediction of willingness were consistent with PU and PEOU (β = 0.53 and 0.53, t = 7.64 and 7.69, *p* < 0.001), and hence, Hypotheses 4 and 5 were supported. Finally, it can be seen that the Behavioral Intention to Use is significantly influenced by the Willingness of the people to use the system (β = 0.78, t = 15.55, *p* < 0.001), and hence, Hypothesis 6 is also strongly and positively supported. It can also be seen that the coefficient of determination (R^2^) value is greater than the standard requirement of 0.10 for all the variables. The regression statistics are depicted in Table 5. With the results of the regression analysis, all seven hypotheses were found to be supported.

## 10. Comparison with Previous Studies

Rashed and Santos conducted a 2010 survey to understand people’s understanding and opinion about using odor as an authentication system [38]. Later that year, Martin Gibbs, an independent scholar, conducted a similar survey by applying and slightly altering Rashed and Santos’ instrument and survey module [7]. In our view, it is of utmost importance to analyze how the acceptance and perception of HBOBT have changed in the last 10 years.

Since these two previous studies were conducted in the same year, using a similar module, they will be considered in one category for comparison with the technology acceptance survey presented in the current work (Table 6).

Several researchers have supported the robustness of TAM in predicting behavioral intentions [68,69]. Furthermore, researchers have also suggested that TAM as a model can be improved by including variables that may be relevant to the context. Keeping in mind the suggestions of other researchers, in the present study, we proposed the OdorTAM model, which is an extension of TAM, comprising additional factors, such as trust and willingness. The findings of the current study are in line with the findings of earlier studies [62,63,68,70]. Although there are studies where it has been found that trust has a significant role in technology acceptance, no direct impact has been found between trust and usage intention [61].

Open comments and opinions
Rashed and Santos [38] and Gibbs [7]
  - “I‘d be curious as to how it functions and how reliable it is. Your base odor can remain the same, however. Fingerprint biometrics have enough issues in functioning consistently; I would miss being shut out of my bank account when I hadn’t had a shower that day yet.”
  - “Odor is not simply an entity and therefore a potential means of recognition; it represents the physical and psychological circumstances of the person being examined (e.g., usage of medications, pregnancy days, stress), and utilizing odor as authentication poses serious ethics concerns.”
  - “For situations where greater safety is required, the use of two or more combined biometric methods could be used.”
  - “Every authentication system that would work without the sampled party’s active involvement is prima facie a system that should be prevented, violating privacy.”
Technology Acceptance Survey (2020)
  - “It’s a good idea to be part of security…and would be used by organizations soon for security purposes…and this way, many unsolved mysteries can be solved.”
  - “I think it can help a lot in various industries and platforms”.
  - “I think it’s a very unique idea, and it can bring a revolution in the field of information security.”
  - “I think odor is a very sensitive and private type of stimulus, which will not become very successful in public spaces.”
  - “Limited chemicals present, not unique enough, easily replicable, not enough in terms of UID.”
  - “This tech might easily get tampered with.”
  - “It is futuristic, but I think it will not be accurate or near to it, but still, it will be profitable to invest in such tech for a better future”.
  - “Perfumes and other factors can hinder this process, in my opinion”.

It is clear from the comparison table how acceptance and perception have increased in comparison to what they were in 2010. There is more knowledge regarding this technique, and people plan to use this biometric technology. Since reports show that there is more widespread access to the internet via devices such as smartphones [71,72], people are comparatively more aware of new techniques and technologies than they were a decade earlier.

## 11. Conclusions

### 11.1. Summary of Results

From the correlation analysis carried out to evaluate the strength and directionality of the OdorTAM hypothesis, we can conclude that the Perceived Usefulness (PU), Perceived Ease of Use (PEOU) and Willingness are quite high, as the Cronbach alpha value is >0.7. However, the results of the analysis show that people do not trust the biometric technique (∝ = 0.59). The reason why people are not trusting the technology could be that they are not aware of it. From the other surveys of the past decade, it is evident that two things have not changed; the first is people’s lack of proper knowledge about technology, and the second is their doubts about its authenticity and security. At the same time, it is noted that people clearly have much more acceptance and will to understand and use the emerging technology. They are also willing to learn about it and recommend it to friends and family as well. Apparently, the respondents had a positive response regarding its usability in cyber forensics and information security. To increase the awareness around this technology, the media and educational institutions must take a step forward in educating the masses. The high values of correlation between the various TAM factors in the current study are in line with the findings of Ahmad and Khan [73] who suggested that, at times, the perceived usefulness plays a mediating role in IT adoption. The findings support the proposed OdorTAM hypotheses.

A total of seven hypotheses were formulated for analyzing the relationships between the various variables. The results of regression analysis show that most of the assumptions are statistically and empirically significant. It was found that the *p*-value corresponding to all variables was less than 0.001, which means that there exists a strong relationship between dependent and independent variable(s) for all the cases. It can be seen from the obtained results that Trust has a strongly positive effect on Perceived Usefulness (PU) (β = 0.78, t = 15.47, *p* < 0.001), and hence, we can say that Hypothesis 1 is supported. The second hypothesis says that a high level of Trust will lead to increased Perceived Ease of Use (PEOU) of a biometric system, and from the regression parameters obtained (β = 0.78, t = 15.40, *p* < 0.001), it is evident that this hypothesis is also supported. The independent variables Trust and Perceived Ease of Use (PEOU) positively influence the dependent variable Perceived Usefulness (β = 0.78 and 0.24, t = 8.83 and 6.96, *p* < 0.001), and therefore, Hypothesis 3 is also strongly supported. The results for the prediction of willingness were consistent with PU and PEOU (β = 0.53 and 0.53, t = 7.64 and 7.69, *p* < 0.001), and therefore, Hypotheses 4 and 5 were supported. Finally, it can be seen that the Behavioral Intention to Use is significantly influenced by the Willingness of the people to use the system (β = 0.78, t = 15.55, *p* < 0.001), and hence, Hypothesis 6 is also strongly and positively supported. It can also be seen that the coefficient of determination (R^2^) value is greater than the standard requirement of 0.10 for all the variables. The OdorTAM model can be surmised as a satisfactory model based on the values of the Cronbach alpha for all the factors, the satisfactory correlation values among the factors and the results of the regression analysis.

The study also has some practical implications. Marketers and developers in the biometric industry can find the study results interesting and useful and work toward further development of these kinds of technologies, which might have a strong position in the market in the future.

### 11.2. Limitations and Future Work

As with any study, there are limitations. There are, for example, some issues related to the survey methodology used. In this study, we utilized a 4-point Likert scale for some of the questions, but it could be argued that there are advantages of using 5- or 7-point scales with a clear midpoint [74]. Another possibility would be to use quantitative values instead of the more qualitative Likert-type scales.

It could also be argued that the features or odors identified in this study are rather vague, and in future work, researchers could try to integrate them into a single value using quantitative techniques, such as fuzzy/gray numbers [75,76,77].

In future work, we would like to explore further how the odor-driven biometric tool can best be exploited in the context of cyber and information security. For instance, in some forensic tests, such as the sniffer dog, smell is used in identifying a particular item or a person. In exploiting smell for this purpose, we can further investigate the specific odor of an individual’s body part, say feet or hand, etc. Accordingly, many forensic related issues could be addressed.

## Figures and Tables

**Table 1:** Distribution of personal and general information.

Question	Response Category	N	%
Gender	Male	85	56.7
	Female	63	42.0
	Other	02	1.30
Age	≤25	100	66.0
	26–50	41	27.3
	51–75	08	5.30
	76–100	01	0.66
Type of Profession	Student	56	37.3
	Technology-Related	27	18.0
	Non-Technology-Related	67	44.6
Did you know about HBOBT in the past?	Yes	30	20.0
	No	106	70.7
	Maybe	14	11.3
Which biometric technology have you seen or used the most in your surroundings?	Iris scanning biometric system	69	46.0
	Finger vein pattern scanning biometric system	112	74.7
	Voice scanning biometric system	48	32.0
	Facial scanning biometric system	82	54.7
	Any other	08	5.30
Which of the following technologies is the most secure according to you?	Human body odor biometric system	28	18.6
	Iris scanning biometric system	99	66.0
	Finger vein pattern scanning biometric system	57	38.0

**Table 2:** Distribution of TAM related responses.

Survey Question	Response Category	N	%
Perceived Usefulness (PU)			
In your opinion, can this biometric technology play an important role in cyber and information security?	Very likely	41	27.3
	Somewhat likely	74	49.3
	Unlikely	26	17.3
	Very unlikely	9	6
This technique can prove to be a better self-identification system.	Very likely	38	25.3
	Somewhat likely	70	46.6
	Unlikely	30	20
	Very unlikely	12	8.00
Perceived Ease of Use (PEOU)			
Do you think it will be easy to use and comfortable?	Very likely	21	14.0
	Somewhat likely	94	62.6
	Unlikely	27	18.0
	Very unlikely	8	5.30
Learning to use the system will be an easy task.	Very likely	20	13.3
	Somewhat likely	90	60.0
	Unlikely	30	20.0
	Very unlikely	10	6.66
Trust (T)			
Do you think that the HBOBT is as reliable as other biometric technologies?	Very likely	19	12.7
	Somewhat likely	80	53.3
	Unlikely	39	26
	Very unlikely	12	8
Can HBOBT improve security and customer privacy?	Very likely	32	21.3
	Somewhat likely	70	46.7
	Unlikely	37	24.67
	Very unlikely	11	7.3
Do you think HBOBT will be invasive and can hinder your privacy in any way?	Very likely	31	20.7
	Somewhat likely	69	46.0
	Unlikely	41	27.3
	Very unlikely	9	6
Willingness (w)			
Do you intend to learn and try out this new biometric technology if available?	Yes	97	64.7
	No	20	16.0
	Maybe	33	22
Would you recommend your family and peers to try out the human body odor biometric system if available?	Yes	83	55.3
	No	24	16.0
	Maybe	43	28.67
Behavioral Intention to Use (BI)			
Assuming you are offered to install a HBOBT at your workplace, would you intend to use it?	Yes	95	63.3
	No	23	15.3
	Maybe	32	21.3
If you had access to the HBOBT in the coming months, would you use it instead of other systems?	Yes	80	53.3
	No	26	17.3
	Maybe	44	29.3

**Table 3:** Mean, standard deviation and reliability of TAM factors.

Survey Question	Mean	Standard Deviation	Cronbach’s Alpha
Perceived Usefulness (PU)			0.78
In your opinion, can this biometric technology play an important role in cyber and information security?	3.01	0.81	
This technique can prove to be a better self-identification system.	2.82	0.85	
Perceived Ease of Use (PEOU)			0.73
Do you think it will be easy to use and comfortable?	2.86	0.71	
Learning to use the system will be an easy task.	2.71	0.79	
Trust (T)			0.59
Do you think that the HBOBT is as reliable as other biometric technologies?	2.71	0.79	
Can HBOBT improve security and customer privacy?	2.82	0.85	
Do you think HBOBT will be invasive and can hinder your privacy in any way?	2.18	0.82	
Willingness (w)			0.78
Do you intend to learn and try out this new biometric technology if available?	2.51	0.72	
Would you recommend your family and peers to try out the human body odor biometric system if available?	2.4	0.74	
Behavioral Intention to Use (BI)			0.56
Assuming you are offered to install a HBOBT at your workplace, would you intend to use it?	2.48	0.52	
If you had access to the HBOBT in the coming months, would you use it instead of other systems?	2.65	0.33	

**Table 4:** Correlation among TAM factors.

	PU	PEOU	Trust	Willingness	Intention to Use
PU	1				
PEOU	0.93170466	1			
Trust	0.80368765	0.78469638	1		
Willingness	0.60095478	0.53478761	0.56918786	1	
Intention to Use	0.53138038	0.50162297	0.53633447	0.94535739	1

**Table 5:** Regression coefficients for extended technology acceptance model.

Independent Variable: Trust (T), Dependent Variable: Perceived Usefulness (PU)					
Model	Unstandardized Coefficients		Standardized Coefficient	t-Value	Significance (*p*-Value)
	B	Standard Error	Beta (β)		
Constant	0.43	0.16		2.63	<0.001
Trust	0.96	0.06	0.78	15.47	
**Independent Variable: Trust (T), Dependent Variable: Perceived Ease of Use (PEOU)**					
Constant	0.57	0.13		4.30	<0.001
Trust	0.72	0.05	0.78	15.40	
**Independent Variable: Trust (T) and Perceived Ease of Use (PEOU), Dependent Variable: Perceived Usefulness (PU)**					
Constant	0.38	0.17		2.21	<0.001
Trust	0.88	0.10	0.78	8.83	
Perceived Ease of Use (PEOU)	0.62	0.09	0.24	6.96	
**Independent Variable: Perceived Usefulness (PU), Dependent Variable: Willingness (w)**					
Constant	1.08	0.18		5.87	<0.001
Perceived Usefulness (PU)	0.47	0.06	0.53	7.64	
**Independent Variable: Perceived Ease of Use (PEOU), Dependent Variable: Willingness (w)**					
Constant	1.45	0.17		8.16	<0.001
Perceived Ease of Use (PEOU)	0.53	0.07	0.53	7.69	
**Independent Variable: Willingness (w), Dependent Variable: Behavioral Intention to Use (BI)**					
Constant	1.77 × 10^−15^	0.16		1.10 × 10^−14^	<0.001
Willingness (w)	0.95	0.06	0.78	15.55	

**Table 6:** Comparison of acceptance of human-body-odor-based technique.

Comparison Factors	Rashed and Santos [38] and Gibbs [7]	Technology Acceptance Survey (2020)
N	84	150
Age interval with maximum respondents	21–26 54%	21–30 67%
Opinion about ease of use	Easy to use—67%	Very likely—14%
		Somewhat likely—63.3%
Opinion about improved security and privacy	Improves security—71%	Very likely—21.3%
		Somewhat likely—46.7%
Intention to use	Yes—42%	Yes—64.7%
Any past knowledge	Yes—10%	Yes—30.3%
